# Neural Correlates of Multisensory Integration for Feedback Stabilization of the Wrist

**DOI:** 10.3389/fnint.2022.815750

**Published:** 2022-05-06

**Authors:** Aaron J. Suminski, Raymond C. Doudlah, Robert A. Scheidt

**Affiliations:** ^1^Department of Biomedical Engineering, Marquette University, Milwaukee, WI, United States; ^2^Department of Neurological Surgery, University of Wisconsin-Madison, Madison, WI, United States; ^3^Department of Biomedical Engineering, University of Wisconsin-Madison, Madison, WI, United States; ^4^Department of Neuroscience, University of Wisconsin-Madison, Madison, WI, United States

**Keywords:** fMRI, motor control, vision, proprioception, error correction (EC)

## Abstract

Robust control of action relies on the ability to perceive, integrate, and act on information from multiple sensory modalities including vision and proprioception. How does the brain combine sensory information to regulate ongoing mechanical interactions between the body and its physical environment? Some behavioral studies suggest that the rules governing multisensory integration for action may differ from the maximum likelihood estimation rules that appear to govern multisensory integration for many perceptual tasks. We used functional magnetic resonance (MR) imaging techniques, a MR-compatible robot, and a multisensory feedback control task to test that hypothesis by investigating how neural mechanisms involved in regulating hand position against mechanical perturbation respond to the presence and fidelity of visual and proprioceptive information. Healthy human subjects rested supine in a MR scanner and stabilized their wrist against constant or pseudo-random torque perturbations imposed by the robot. These two stabilization tasks were performed under three visual feedback conditions: “No-vision”: Subjects had to rely solely on proprioceptive feedback; “true-vision”: visual cursor and hand motions were congruent; and “random-vision”: cursor and hand motions were uncorrelated in time. Behaviorally, performance errors accumulated more quickly during trials wherein visual feedback was absent or incongruous. We analyzed blood-oxygenation level-dependent (BOLD) signal fluctuations to compare task-related activations in a cerebello-thalamo-cortical neural circuit previously linked with feedback stabilization of the hand. Activation in this network varied systematically depending on the presence and fidelity of visual feedback of task performance. Addition of task related visual information caused activations in the cerebello-thalamo-cortical network to expand into neighboring brain regions. Specific loci and intensity of expanded activity depended on the fidelity of visual feedback. Remarkably, BOLD signal fluctuations within these regions correlated strongly with the time series of proprioceptive errors—but not visual errors—when the fidelity of visual feedback was poor, even though visual and hand motions had similar variability characteristics. These results provide insight into the neural control of the body’s physical interactions with its environment, rejecting the standard Gaussian cue combination model of multisensory integration in favor of models that account for causal structure in the sensory feedback.

## Introduction

Stabilizing hand-held objects is an important behavior in everyday life. Despite decades of study, it remains unclear how the brain uses sensory information to control manual interactions with physical objects. Stabilizing a hand-held object like a glass of water relies heavily on visual feedback to determine, for example, the tilt of the water relative to the rim. Other actions like stabilizing a car’s steering wheel are dominated by proprioceptive feedback because maneuvering through traffic requires visual attention to be focused on other vehicles. Still other activities require flexible patterns of multisensory control, where the relative importance of visual and proprioceptive feedback varies as the dynamic demands of the task change. For example, a restaurant server uses visual and proprioceptive cues to stabilize a hand-held serving tray when removing one of several dishes to be served, but likely uses proprioception alone to stabilize the tray when delivering a plate to table because visual attention is required to avoid table-top obstacles such as glasses and silverware. How does the brain combine multiple sources of sensory information for ongoing limb stabilization? This paper addresses that question within the context of a limb stabilization task we previously used to study electromyographic and neural correlates of proprioceptive feedback control (Suminski et al., 2007a). We now ask how the presence and fidelity of visual feedback impacts the neural processing of proprioceptive and visual signals related to feedback stabilization of the wrist against uncertain environmental loads.

For decades, there has been debate about how the brain integrates information from the different senses to estimate limb state for perception (Tillery et al., 1991; Ernst and Banks, 2002; van Beers et al., 2002; Ernst, 2006; Reuschel et al., 2010) and action (Soechting and Flanders, 1989; Henriques and Crawford, 2002; Körding and Wolpert, 2004; Scheidt et al., 2005; Bagesteiro et al., 2006; Judkins and Scheidt, 2014; Crevecoeur et al., 2016). As one example, Ernst and Banks (2002) presented human subjects with sequential pairs of “raised ridge” stimuli that they could view binocularly and/or grasp with the index finger and thumb (Ernst and Banks, 2002). Each presentation of visual and/or mechanical stimuli lasted for 1 s and could vary in height. Noise was sometimes added to the visual display to vary its reliability. The subject’s task was to indicate which of the paired stimuli (first or second) was apparently taller. The resulting data suggested that the combination of sensory cues in the presence of noise was well-described by an integration rule based on *Maximum Likelihood Estimation* (MLE), which proposes that the brain combines information from each sensory modality in a way that minimizes uncertainty (variance) in a unified multisensory state estimate:


(1) $$S_{MS}\left(t\right)=\sum_{i}w_{i}S_{i}\left(t\right),\text{with}w_{i}=\frac{\sigma_{i}^{-2}}{\sum_{j}\sigma_{j}^{-2}}.$$

In this model, the multisensory percept *S*_*MS*_(*t*) is a combination of evidence provided by sensory cues *S_i_*(*t*) weighted in inverse proportion to the cues’ uncertainties $\sigma_{i}^{-2}$. The more reliable the signal, the more it contributes to the multisensory estimate of the state. By contrast, others have suggested that the brain combines sensory cues for generating actions using context-specific weighting schemes that may not strictly adhere to the “static” integration rule described by Equation 1. For example, in one study of goal-directed reaching, Sober and Sabes (2003) reported evidence that multisensory integration rules vary depending on what aspect of movement is being planned. Limb position estimation for planning the direction and extent of a goal-directed reach appears to rely mostly on visual feedback, whereas limb position estimation for computing requisite motor commands appears to be biased toward proprioceptive information (Sober and Sabes, 2003). While such findings do not outright contradict the conclusions of Ernst and Banks (2002), they do suggest that multisensory integration in the estimation of limb state for action may well be context-dependent, adjusting dynamically even within the early stages of planning and executing a single goal-directed action.

In another relevant study, Judkins and Scheidt (2014) used a simple virtual reality display and a hand-held robotic handle to examine sensorimotor adaptation of goal-directed reaching in response to robotic (physical) and/or virtual (visual) spring-like loads that varied randomly from one trial to the next. The virtual load was driven by forces applied to the robot’s handle and thus, the cursor’s motion could differ from that of the physical load (i.e., the handle) if the simulated spring constants differed. This decoupling of the physical and virtual responses to perturbation allowed independent assessment of the influence of visually and proprioceptively perceived performance errors on subsequent movements. Surprisingly, the authors observed complete visual capture in the trial-by-trial updating of goal-directed reaches despite the presence of substantial uncertainty in both the visual and proprioceptive percepts. Based on the experimental data, the authors concluded that multisensory integration for the adaptive control of reaching did not conform to predictions of a MLE model, which instead predicted incomplete visual capture (i.e., proprioceptive contributions in the presence of a moderate visual bias) (Judkins and Scheidt, 2014). One possible explanation for why a MLE of sensory integration might fail to describe multisensory integration for the trial-by-trial correction of movement errors is that the movements in that study were fast and performance feedback was fleeting at the moment of target capture. By contrast, subjects in perceptual tasks typically have much more time to explore the stimuli and to make a perceptual decision. Severe limitations in the amount of time available for multisensory integration could constrain the neural computations responsible for integrating sensory feedback for the control of action, forcing the brain to choose one modality over the other based on factors other than just the relative reliability of the sensory cues (cf., a summary of Crevecoeur et al., 2016 in “DISCUSSION”). Another possibility is that unimodal sensory capture arises when the several feedback sources differ to such an extent that they no longer are interpreted as deriving from a common source, again forcing the brain to choose one modality over the other to drive goal-directed actions. The absence of integration is predicted by *Bayesian Causal Inference* (BCI) models of perception (e.g., Körding et al., 2007; Debats et al., 2017), which only integrate multimodal sensory cues as in Equation 1 if they have a common cause but keeps them segregated if they have independent causes.

Here, we probe the neural mechanisms of multimodal sensorimotor control in a task (wrist position stabilization) that allows prolonged time-on-task while also permitting independent manipulation of visual and proprioceptive feedback of task performance. Subjects reclined in a magnetic resonance (MR) scanner and underwent functional MR imaging (fMRI) while stabilizing their hand against two forms of robotic perturbations—constant and random wrist torques—using three different forms of real-time sensory feedback: no visual feedback (i.e., proprioception only), veridical visual feedback wherein a visual cursor tracked hand motion faithfully, and a random vision condition wherein cursor motion was uncorrelated with actual hand motion. We analyzed correlations between blood-oxygenation level-dependent (BOLD) signal fluctuations and time series of visual and proprioceptive performance feedback to probe how the presence and fidelity of visual feedback of task performance impacts task-related activations in a cerebello-thalamo-cortical neural circuit previously associated with mechanical stabilization of the upper extremity (cf, Suminski et al., 2007a). We examined the extent to which the neural correlates of multisensory integration for control align with predictions of the MLE and BCI models of perception. The results advance a fundamental understanding of how sensory context impacts information processing in the neural circuits responsible for feedback stabilization of the hand against unpredictable environmental perturbations.

## Materials and Methods

Twelve right-handed adults (4 female) participated in this study. Subjects were between the ages of 19 and 48 years (27.5 ± 8.4 year; mean ± 1 SD, here and elsewhere). All subjects were strongly right-handed according to the Edinburgh Handedness Inventory (Oldfield, 1971). Exclusion criteria included: Significant neurological, psychiatric or other medical history, currently taking psychoactive medications, and additional exclusion criteria specific to MR scanning: Ferrous objects within the body, weight inappropriate for height, pregnancy, low visual acuity, or a history of claustrophobia. No participants were excluded from this study based on these criteria. Written informed consent was obtained from each subject in accord with the Declaration of Helsinki and with the institutional guidelines of Marquette University and the Medical College of Wisconsin.

### Experimental Procedure

**FIGURE 1:**
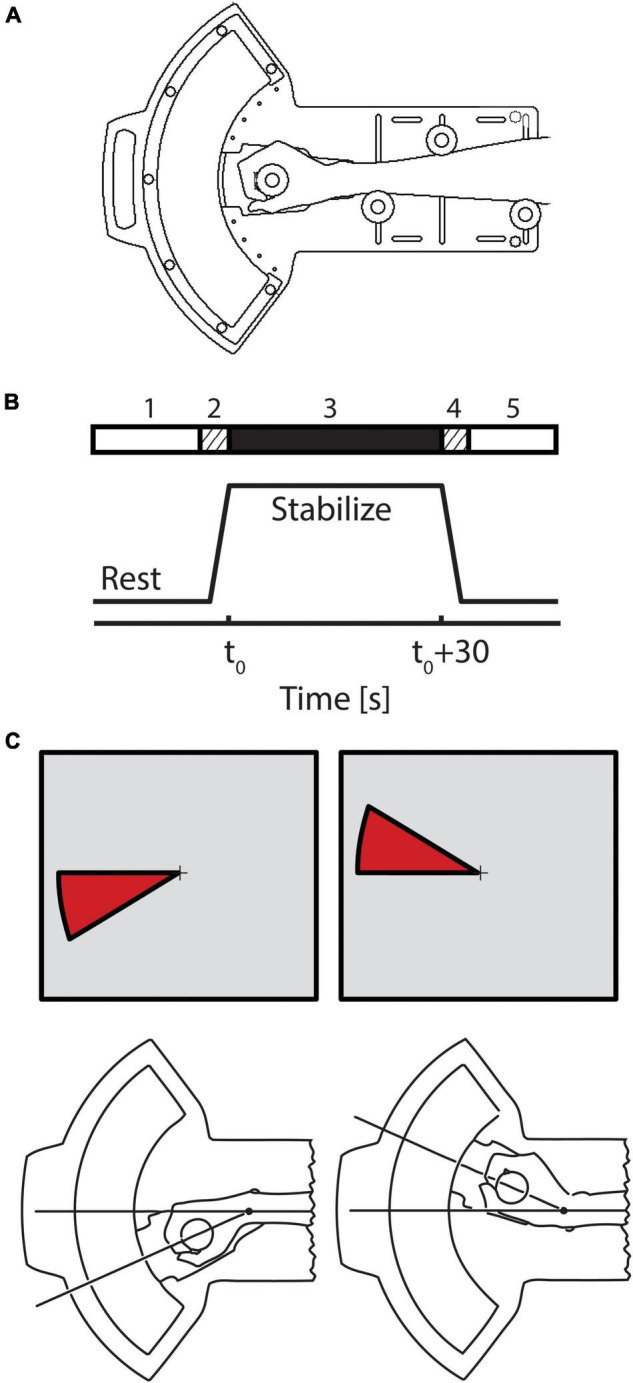
**(A)** Schematic representation of the 1 degree of freedom pneumatic manipulandum illustrating how the subject interfaces to the device. **(B)** A single trial was conducted in 5 phases. During the 30 s prior to stabilization (phase 1), the subject was instructed to relax while the robot held the hand in a comfortable posture of 40° flexion. Three seconds prior to the start of stabilization (phase 2), the robot moved the relaxed hand to the target posture (20° flexion) and held it there until the onset of the stabilization period. During stabilization periods (phase 3), subjects were instructed to maintain their wrist position at 20° flexion. At the end of the stabilization period, the subject was instructed to relax, and the robot moved the passive hand to its resting position at 40° flexion (phase 4) after which the subject rested (phase 5) in preparation for the next trial. **(C)** On select trials, visual feedback of wrist angle was provided *via* a wedge-shaped cursor projected onto a vertical display screen. A small fixation point (cross hairs) was visible in the middle of the screen throughout the entire experiment.

Subjects rested supine in a 1.5T General Electric Signa scanner (General Electric Healthcare, Milwaukee, WI) at Froedtert Memorial Lutheran Hospital in Milwaukee, Wisconsin. The scanner was equipped with a 3-axis local gradient head coil and an elliptical endcapped quadrature radiofrequency coil. The subjects’ heads were constrained by foam padding to reduce motion inside the head coil. With arms at their sides, subjects grasped the handle of a MR-compatible, 1 degree-of-freedom robotic manipulandum with their right hands (Figure 1A). The handle’s axis of rotation was aligned with that of the wrist. The frame of the device was secured to both the subject’s forearm and the inner wall of the scanner bore for support. The robot includes a pneumatic actuator that exerts computer-controlled torques about the wrist. Analog measurements of pressure within the actuator were amplified and low-pass filtered with a cutoff frequency of 20 Hz. The torque applied at the wrist joint was computed based on the pressure in the actuator and the moment arm of the device. Robotic signal processing and control was performed at a rate of 1,000 samples per second. Additional details of the robotic system’s design, performance and MR-compatibility are described elsewhere (Suminski et al., 2007b).

Each subject performed a series of wrist stabilization tasks while simultaneously undergoing fMRI scanning. A single stabilization trial was conducted in 5 phases (Figure 1B). Phase 1: During the 30 s prior to stabilization onset, the subject was instructed to relax while the robot held the hand in a comfortable resting posture *θ_*r*_* (40° flexion). Phase 2: 3 s prior to the start of stabilization, the robot moved the relaxed hand to the target posture (20° flexion) and held it there until stabilization onset. Phase 3: During the stabilization period itself (30 s in duration), subjects were instructed to maintain wrist position against one of two types of extensor torque loads. In one, the robot was programmed to apply a predictable, *constant torque* (***CT***, mean = 1.2 Nm). In the other, the device applied *pseudo-random torques* (***RT***) consisting of band-limited, Gaussian, “white” noise (1.2 ± 1.1 Nm) having the same average extensor torque as the constant perturbation and a low-pass cutoff frequency of 1.6 Hz. Phase 4: At the end of the stabilization period, the subject was instructed to relax as the robot moved the passive hand back to its resting position at 40° flexion. Phase 5: the subject rested until the start of the next trial.

Although direct view of the wrist was precluded, a wedge-shaped cursor (Figure 1C) was sometimes projected onto a screen at the subject’s feet using a back-projection LCD projector. This cursor represented error between current and desired wrist angles. Subjects viewed the screen using prism glasses, which allowed them to see the visual feedback while lying on their back as if it were displayed on a screen placed directly in front of them. Subjects were provided with one of three types of visual feedback during stabilization: *true vision* (***TV***), *pseudo-random vision* (***RV***), and *no vision* (***NV***). Accurate real-time feedback of wrist angle relative to the target angle was provided in the ***TV*** condition. In the ***RV*** condition, a “surrogate” band-limited Gaussian noise signal replaced the actual wrist angle for computing real-time location of cursor feedback. Surrogate visual feedback was matched to the hand displacement profiles under ***RT*** perturbation both in range and spectral content, but was constructed to include no significant temporal correlation with the pseudo-random torque perturbation sequence. This construction ultimately allowed for independent assessment of neural correlates of physical (proprioceptive) and visual feedback of performance errors. In the ***NV*** condition, no cursor wedge was displayed. In all cases however, a thin, stationary fixation target was displayed in the center of the display screen in an attempt to minimize extraneous eye movements.

Each subject participated in a single, blocked-design experiment requiring alternating periods of rest and active stabilization. Each subject performed 10 functional imaging “runs,” which included each of the 6 trial types (2 torque × 3 visual conditions) presented one time per run in pseudo-random order. During each run, whole-brain images were acquired using a single-shot, blipped gradient-echo echo-planar pulse sequence (19 contiguous sagittal 7-mm slices, TE = 40 ms, TR = 2.5 s, 90° flip angle, FOV = 24 cm, 64 × 64 matrix, 3.75-mm in-plane resolution). Blood-oxygenation level-dependent (BOLD) signal contrast was used to image hemodynamic-related changes evoked by stabilization in the 6 trial conditions. High-resolution 3D spoiled gradient recalled at steady-state T1-weighted anatomic images also were collected prior to functional imaging for subsequent anatomic localization and co-registration of the functional images (TE = 5 ms, TR = 24 ms, 40° flip angle, slice thickness = 1.2 mm, FOV = 24 cm, 256 × 192 matrix).

### Behavioral Data Analysis

Time series of wrist joint angle and joint angular velocity were low-pass filtered at a cutoff frequency of 10 Hz. Stabilization was evaluated using several kinematic performance measures. We computed *objective stabilization error* ε_*o*_(*nT*) as the difference between the actual and desired (target) hand positions:


(2) $$\varepsilon_{o}\left(nT\right)=\theta \left(nT\right)-\theta_{t}$$

where θ_*t*_ is the targets wrist angle (20° flexion) and θ(*nT*) is the instantaneous wrist angle at sample instant *nT*. To compare objective performance across stabilization conditions, we then computed the root mean square (RMS) value of this objective error time series throughout each 30-s trial (i.e., *RMS*(ε_*o*_(*nT*))).

We quantified *drift* in the instantaneous joint angle equilibrium position by fitting a first-order polynomial to the joint angle time series data over the final 20 s of each trial. We only considered the final portion of each trial to avoid start-up transients that were visible within the first 5 s on some trials. Drift was considered significant in those trials where the slope of the regression line was statistically different from zero. This polynomial defined the subjective wrist target angle θ_*s*_(*nT*) as the instantaneous reference angle about which small corrections were observed. We used θ_*s*_(*nT*) to estimate a *subjective stabilization error* ε_*s*_(*nT*) [i.e., the instantaneous deviations of the wrist about θ_*s*_(*nT*)]:


(3) $$\varepsilon_{s}\left(nT\right)=\theta \left(nT\right)-\theta_{s}\left(nT\right).$$

We compared subjective performance across stabilization conditions using the *RMS*(ε_*s*_(*nT*)) value computed in each trial.

We constructed an estimate of subjective wrist *state estimation errors* ε_*q*_(*nT*) during phases 2 through 4 of each trial (i.e., during stabilization as well as during the preceding and following passive movement phases) under the assumptions that during trial phases 2 and 4, passive movement of the wrist induced a discrepancy between actual limb position and the angle expected given the recent history of motor output (resting angle θ_*r*_ = 40° flexion; target angle θ_*t*_ = 20° flexion), whereas during phase 3, state estimation errors would arise from load-induced deviations from the subjective target angle θ_*s*_(*nT*). Specifically,


(4) εq(nT)={θ(nT)-θr;phase 2εs(nT);phase 3θ(nT)-θt;phase 4

The time series of ε_*q*_(*nT*) were used to compute RMS values of limb state estimation errors on a moment-by-moment basis [i.e., within each 2.5 s (2,500 sample) integration window, thereby emulating the temporal sampling of the functional imaging pulse sequence, TR], thus obtaining *RMS*_*TR*_(ε_*q*_(*m*)), where m is an index running from 1 to the total number of TR sampling intervals spanning phases 2 through 4 of the trial.

We quantified *visual stabilization error* ε_*v*_(*nT*) during trial phase 3 as the difference between the instantaneous visual representation of wrist angle and the target wrist angle (i.e., the angular size of the wedge-shaped cursor). Because visual feedback faithfully tracked wrist angle during trials with ***TV*** feedback and because visual feedback was absent during ***NV*** trials, ε_*v*_(*nT*) was quantifiably distinct from ε_*s*_(*nT*) only during ***RV*** trials. For subsequent use in functional neuroimage analysis, we computed the RMS value of ε_*v*_(*nT*) within 2.5 s integration windows to emulate the temporal sampling of the functional imaging pulse sequence, thus obtaining *RMS*_*TR*_(ε_*v*_(*m*)).

### Statistical Inference for Behavioral Performance Measures

Objective RMS stabilization errors and the unsigned magnitude of positional drift were averaged within subject by trial type. Individual 2-way repeated measures ANOVA assessed differences in stabilization error and drift due to the two factors: Torque perturbation type (***RT***, ***CT***) and visual feedback type (***TV***, ***NV***, or ***RV***). *Post-hoc* Tukey *t*-tests were used to identify the source of significant main and interaction effects. Statistical testing was carried out within the Minitab computing environment (Minitab, Inc., State College, PA). Effects were considered statistically significant at the α = 0.05 level.

### Functional MR Imaging Data Analysis

Functional MR images were generated and analyzed within the Analysis of Functional NeuroImages (AFNI) software package (Cox, 1996). During each run, a total of 156 images were collected at each voxel yielding a total run duration of 6.5 min. The three images at the beginning and end of each run were discarded to allow for equilibration of the magnetic field. For each subject, the resulting 150 point time series from each of the 10 imaging runs were first concatenated into one large dataset. We then used an interactive, linear, least squares method to align the images in three-dimensional space to counteract the effects of head motion. Registration yielded 6 movement indices per functional imaging run. The across-subjects average head movement for the rotation indices were 0.63 ± 0.4°, 0.29 ± 0.16°, and 0.55 ± 0.44° (rotations in the superior-inferior, anterior-posterior, and left-right planes, respectively); average translational head movement were 0.95 ± 0.49 mm, 0.43 ± 0.28 mm, and 0.53 ± 0.24 mm (translation in the superior-inferior, anterior-posterior, and left-right direction, respectively). No subjects or trials were excluded from further analysis due to head motions because none were found to exceed ½ the smallest voxel dimension.

In a previous study (Suminski et al., 2007a), we asked subjects to stabilize their wrists against robotic perturbations in the absence of ongoing visual feedback and used a hierarchical multilinear regression technique to identify BOLD signal correlates of error corrections that operate over longer (trial-by-trial) and shorter (TR-by-TR) time scales. Here, we extended that approach to determine how the neural mechanisms regulating hand position respond to the presence and fidelity of visual feedback of ongoing performance. To do so, we modeled BOLD signal fluctuations within each voxel as a combination of three independent sources of variability: (1) nuisance variables common to fMRI data collection (i.e., head motion and baseline BOLD signal drift); (2) factors generally related to the performance of the visuomotor stabilization task that do not change from one trial to the next; and (3) factors related to both visual and proprioceptive performances errors that changed from TR-to-TR. In our analysis, unmodeled signal variations that remained after an initial block-wise analysis (*Stage 1 Regression—Baseline Noise Model and Block-by-Block Effects*) became the input to a subsequent TR-by-TR analysis focusing on moment by moment changes in task performance (*Stage 2 Regression—TR-by-TR Effects*).

### Stage 1 Regression—Baseline Noise Model and Block-by-Block Effects

We performed an initial voxel-wise multiple linear regression analysis that served two purposes: (1) To remove from the fMRI dataset all BOLD signal modulations correlated with nuisance cofactors such as head motion and baseline drift; and (2) to identify fMRI signal fluctuations that were related generally to execution of the wrist stabilization task under each testing condition but did not vary in a manner reflecting moment-by-moment task performance. The baseline noise model included the six time series of head motion indices obtained from the spatial registration process (sample interval = 1 TR). By including these subject- and run-specific nuisance factors in the multilinear regression, we reduced the likelihood of false positive results due to stimulus-correlated motion. Next, we defined an input reference function for each of the six stabilization conditions to model general task-dependent effects. These time series were assigned a value of 1 during their respective stabilization periods and 0 otherwise. Each of these reference functions was created separately for each run performed by each participant, reflecting the pseudo-randomized presentation order of task conditions across runs and participants. Each time series was then convolved with a γ-variate function to model the temporal filtering properties of the hemodynamic response.

### Stage 2 Regression—TR-by-TR Effects

The purpose of the Stage 2 analysis was to identify BOLD signal variations that correlated significantly with the moment-by-moment (TR-by-TR) changes in wrist or cursor positioning errors [i.e., *RMS*_*TR*_(ε_*q*_) and *RMS*_*TR*_(ε_*v*_), respectively]. We restricted this event-related analysis to include trials only wherein subjects experienced persistent physical perturbations and subsequent error corrections (i.e., ***RT*** conditions) or visual feedback that varied moment by moment (i.e., ***TV*** and ***RV*** conditions). Therefore, ***CTTV*** and ***CTNV*** trials were not included in this analysis because performance errors were relatively constant throughout the trial. Note that in ***RTTV*** trials, proprioceptive and visual performance errors were perfectly correlated whereas in ***RTRV*** trials, proprioceptive and visual performance errors were minimally correlated.

We therefore created a total of four reference functions to explore the relationship between BOLD fluctuations and behavioral errors. Two of these time series were derived from the ***RTNV*** and ***RTRV*** conditions on a TR-by-TR basis; they quantified performance errors that could be sensed proprioceptively. The time series derived from the ***CTRV*** and ***RTRV*** conditions quantified TR-by-TR variations in visual errors that were distinct from proprioceptive errors. The last reference function was derived from the ***RTTV*** condition, and it jointly represented both proprioceptive and visual errors when they were highly correlated. The proprioceptive and visuomotor reference function values at each TR sampling instant were defined, respectively, by the *RMS*_*TR*_(ε_*q*_) and *RMS*_*TR*_(ε_*v*_) values computed during the corresponding 2.5 s TR sampling period. Each of these reference functions was created separately for each run performed by each participant, and then convolved with a γ-variate function to model the temporal filtering of the hemodynamic response.

### Statistical Inference for Functional MR Imaging Data

Functional images resulting from the hierarchical multilinear regressions were interpolated to obtain a volumetric grid having 1 mm^3^ voxel volumes, co-registered, and then converted into the Talairach stereotaxic coordinate space. To facilitate across-subjects analyses, the normalized functional images were spatially blurred using a 4-mm Gaussian, full-width half-maximum filter to compensate for inter-subject anatomical variability. In all across-subject analyses, a cluster-size and thresholding technique was used to correct for multiple comparisons in the group analysis to reduce type-I inference errors at the α = 0.05 level. We performed a 10,000-iteration Monte-Carlo simulation using the *3dClustSim* tool within AFNI to identify cluster sizes and individual voxel *p*-values appropriate for the Stage 1 block-wise effects analysis (cluster size: 289 μl; individual voxel *p*-value: 0.001). A second 10,000-iteration Monte-Carlo simulation was performed to identify cluster sizes and individual voxel *p*-values appropriate for the Stage 2 event-related analysis (cluster size: 505 μl; individual voxel *p*-value: 0.005). The use of a less conservative individual voxel probability value in the TR-by-TR effects analysis was justified because the BOLD signal fluctuations of interest were small and embedded within the residuals of the Stage 1 analysis. The locations of activated regions in the group statistical parametric maps were obtained using the integrated atlas within AFNI. Surface based representations of cortical activations were visualized using CARET (Van Essen et al., 2001).

For the analysis of BOLD signal activations on a longer (block-by-block) time scale, we analyzed the functional neuroimaging data in a manner similar to our analysis of behavioral data: we used a 3-way, mixed-model, repeated measures ANOVA (treating subjects as a random factor) to identify voxel clusters exhibiting BOLD signal fluctuations that correlated systematically with variations in load type, visual feedback condition, and the interaction of these two factors. First, we used *post-hoc t*-tests to identify patterns of neural activity that were related—in a general sense—to the active compensation for wrist position errors (the “Error Correction” contrast). We did so by contrasting BOLD signal changes (relative to rest) across the task conditions requiring stabilization against random vs. constant torque perturbations (i.e., the three ***RT*** conditions vs. the three ***CT*** conditions). Second, we used the results of *post-hoc t*-tests to visualize the neural mechanisms responding to visual motion of the cursor during stabilization (the “Visual Motion” contrast). Here, we contrasted the {***RTTV***, ***RTRV***} conditions vs. the ***RTNV*** condition, ignoring all ***CT*** conditions wherein motion of the cursor was absent (***CTNV***), negligible (***CTTV***), or obviously discrepant (***CTRV***). Finally, we examined the interaction between the two factors by performing a set of three contrasts that explored how neural activities related to error correction are modulated by the presence and fidelity of real-time visual feedback (the “Visual Interaction Effect”). Here, we used three separate *t*-tests to contrast BOLD signal responses across ***RT*** vs. ***CT*** stabilization tasks during the three visual feedback conditions (i.e., ***RTNV-CTNV, RTTV-CTTV*,** and ***RTRV-CTRV***).

Next, motivated by our previous finding that BOLD signal contrast within brain regions involved in the proprioceptive control of wrist position are sensitive to performance errors on a moment-by-moment basis (Suminski et al., 2007a), we used a 2 way, mixed-model, repeated measures ANOVA (treating subjects as a random factor) to identify voxel clusters exhibiting BOLD signal fluctuations that correlated significantly with TR-by-TR changes in performance errors. This analysis of error corrections on a short, moment-by-moment time scale used the four separate and orthogonal reference functions that captured the TR-by-TR variations in hand and cursor motion as described earlier (*Stage 2 Regressions—TR-by-TR Effects*). For this analysis, we applied *post-hoc*, voxel-wise *t*-tests (against 0) to the regression coefficients for each reference function to identify BOLD signal correlates of TR-by-TR changes in performance errors sensed proprioceptively (***RTNV*** and ***RTRV***), visually (***CTRV*** and ***RTRV***), or jointly (***RTTV***). Finally, we planned two additional contrasts to explore how neural activities related to the TR-by-TR correction of proprioceptive errors are modulated by the presence and fidelity of real-time visual feedback (i.e., separate *t*-tests performing the ***RTTV-RTNV*** and ***RTRV-RTNV*** contrasts.

## Results

### Behavioral Correlates of Sensorimotor Stabilization

**FIGURE 2:**
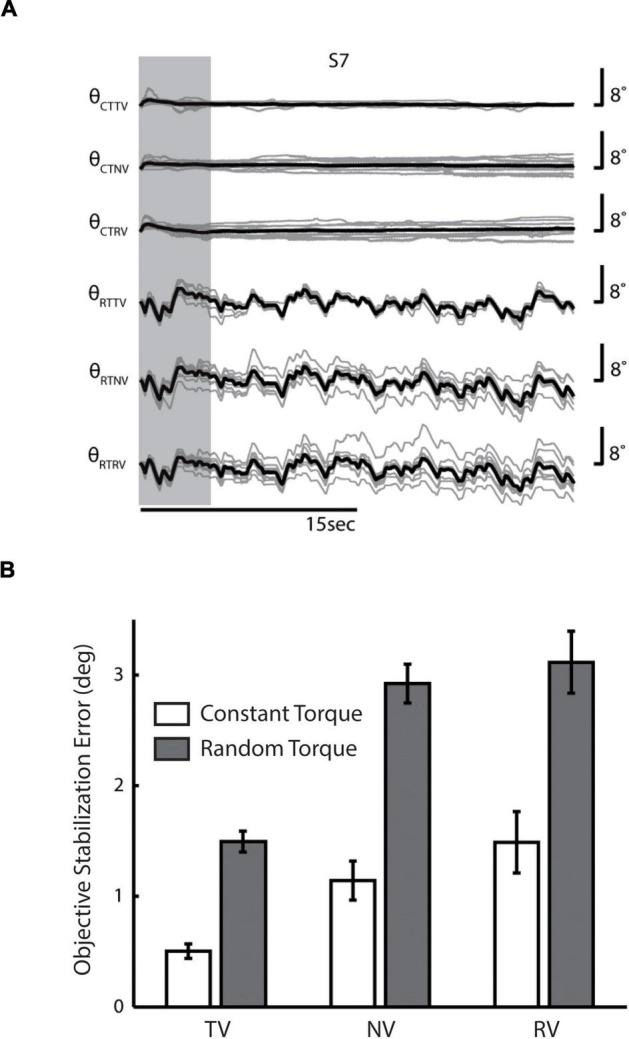
**(A)** Wrist angle error θ for a representative single subject (S7) in each of the six combinations of two environmental loads (***CT***, constant torque; ***RT***, random torque) and three sensory feedback conditions (***TV*,** true vision; ***NV***, no vision; ***RV***, random vision). Light gray lines indicate the subject’s performance on individual trials, whereas heavy black lines denote the mean performance of the subject across trials. Vertical gray band: initial portion of data where startup transients were frequently observed. **(B)** Population statistics—comparison of averaged RMS wrist angle errors across feedback conditions (***CT***, white bars; ***RT***, shaded bars). Error bars indicate the 95% confidence interval about the mean (i.e., ± 1.96 * standard error).

We first sought to determine the extent to which the presence and fidelity of visual feedback and differences in load type might have elicited differences in performance during stabilization. Because torque perturbations were biased into wrist extension in all task conditions, subjects were required to actively engage in the task to perform with any degree of success. If they were to “give up,” the wrist would be driven into the robot’s mechanical limits at 30° extension. Because no wrist angle trajectories were observed to reach and remain at 30° extension, we infer that all of the participants performed in a task-appropriate manner on every trial. Nevertheless, wrist angle deviations from the target were variably compensated both within and between trials; subjects were able to recover the desired reference position only on average across many trials (Figure 2A).

Linear regression found significant drift in wrist angle as a function of time in the vast majority of trials (***RTTV:*** 79% of trials, with the absolute magnitude of drift averaging 0.05 ± 0.03°/s; ***RTNV:*** 98%, 0.14 ± 0.06°/s; ***RTRV:*** 98%, 0.14 ± 0.06°/s; ***CTTV:*** 76%, 0.01 ± 0.01°/s; ***CTNV:*** 77%, 0.03 ± 0.02°/s; ***CTRV:*** 79%, 0.03 ± 0.02°/s). In all cases, drift was evenly distributed about the target angle and varied randomly from one trial to the next. The magnitude of positional drift varied both by load type [*F*_(1, 60)_ = 135.7, *p* < 0.0005] and by visual feedback condition [*F*_(2, 60)_ = 19.8, *p* < 0.0005]. The interaction between these factors was also significant [*F*_(2, 60)_ = 8.9, *p* < 0.0005]. *Post-hoc t*-tests found that the magnitude of drift was greatest when subjects were perturbed by pseudo-random torques without reliable visual performance feedback (***RTNV*** and ***RTRV* vs. *all other cases***; *p* < 0.0005). In the ***TV*** cases, drift was approximately one third that observed in the ***NV*** and ***RV*** cases, regardless of perturbation type. The magnitude of drift observed here is consistent with that reported in an earlier study of limb stabilization without concurrent visual feedback of performance (Suminski et al., 2007a).

**FIGURE 3:**
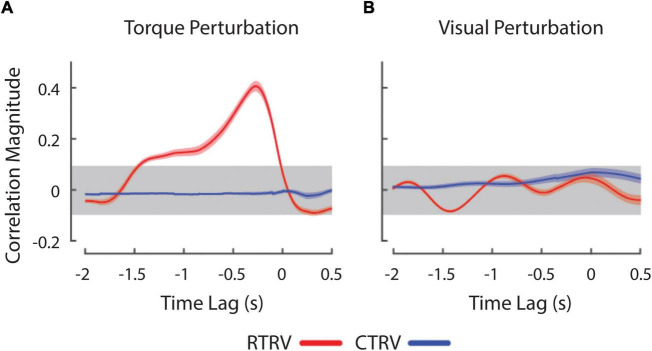
Cross-correlation between wrist angle and experimental perturbations during ***RTRV*** (red lines) and ***CTRV*** (blue lines) trials. **(A)** Cross-correlation between wrist angle and the ***RT*** perturbation averaged across subjects. **(B)** Cross-correlation between the wrist angle and ***RV*** perturbation averaged across subjects. Peaks at negative lags denote conditions where changes in the perturbation precede movement of the wrist in time. The horizontal gray band represents an empirical estimation of the spurious correlation associated with the time series. Error envelopes indicate 1 standard error about the mean correlation.

Next, we investigated the effects of load type and visual feedback on the RMS objective stabilization performance. We found significant main effects of both load type and visual feedback condition on RMS objective stabilization performance as shown in Figure 2B [Load Type: *F*_(1, 60)_ = 87.1, *p* < 0.0005; Visual Feedback: *F*_(2, 60)_ = 27.0, *p* < 0.0005]. The interaction between load type and visual feedback condition failed to reach statistical significance. On average, subjects were less able to maintain steady hand posture while being perturbed by band-limited pseudo-random torques than by constant torques (*p* < 0.0005). Relative to the ***TV*** conditions, performance degraded markedly as visual feedback was made less reliable (***RV***: *p* < 0.0005) or was eliminated altogether (***NV***: *p* < 0.0005). We found no difference in performance between the ***NV*** and ***RV*** conditions, raising the possibility that subjects might have ignored the visual feedback provided during both ***RV*** conditions. To investigate this possibility, we computed the cross correlation between objective stabilization error and the torque or visual perturbation on ***CTRV*** and ***RTRV*** trials (Figure 3). As expected, we observed strong, positive cross-correlations between torque perturbation and stabilization error on ***RTRV*** trials at a time lag averaging –273 ms (i.e., with torque perturbations leading errors by about 1/4 s). In contrast, we found no evidence of correlation between objective stabilization errors and the visual perturbation on ***CTRV*** and ***RTRV*** trials. This supports the supposition that subjects severely discounted visual feedback on these two trial types, even though the magnitude and spectral content of the visual error signals were similar to those in the ***RTTV*** case, which exhibited much smaller performance errors.

### Neural Correlates of Sensorimotor Stabilization

#### Stage 1 Analyses: Block-by-Block Effects

**FIGURE 4:**
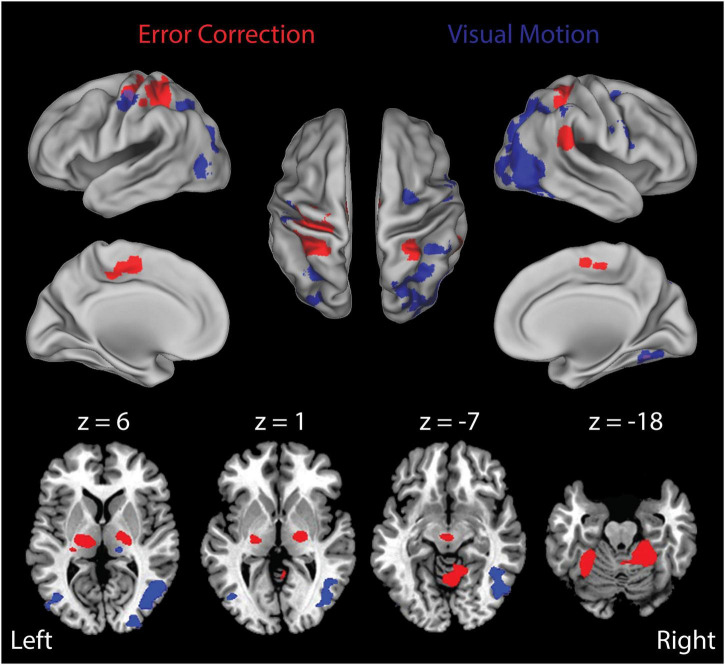
Results of the Stage 1 voxel-wise multiple linear regression: block-by-block effects. BOLD signal correlates of ongoing error correction and visual motion are depicted in separate arbitrary colors that should not be interpreted as a “heat map” signal intensity. Red: functional activation maps for the study population showing the regions of interest (ROIs) that exhibited significantly enhanced activation during stabilization against random environmental torques relative to constant torques (***RT*** > ***CT***) (i.e., the “Error Correction” contrast). This contrast highlights activities related to ongoing error correction (***RT*** trials) above and beyond those related more generally to the wrist stabilization task (***CT*** trials). We observed prominent activation within a cerebello-thalamo-cortical network when stabilizing against unpredictable loads as opposed to predictable loads. Blue: ROIs that exhibited enhanced activation during stabilization in ***RT*** trials with visual motion (the ***RTTV*** and ***RTRV*** cases) relative to stabilization in ***RT*** trials with no visual motion (the ***RTNV*** case) (i.e., the “Visual Motion” contrast). We observed prominent activation within secondary- and higher-order visual processing areas including middle/inferior occipital gyrus, middle temporal gyrus, fusiform gyrus, inferior parietal lobule and premotor cortex. Colored areas indicate regions that were shown to be significantly active in these contrasts at the *p* < 0.05 level of significance (corrected for multiple comparisons). Top: activations mapped onto inflated representations of the cerebral hemispheres; bottom: subcortical activations in the basal ganglia and thalamus (left: *z* = 6; center-left: *z* = 1) and anterior cerebellar cortex (center-right: *z* = –7; right: *z* = –18).

As noted above, participants never “gave up” and each engaged in active feedback stabilization of the wrist on every trial. Figure 4 presents the results of our Stage 1 block-by-block analyses, wherein we separately examined the effects of load type and visual feedback conditions on the neural mechanisms engaged during wrist stabilization. Shown in red are regions of interest (ROIs) that exhibited enhanced BOLD signal activation in block conditions requiring active stabilization against ***RT*** perturbations that elicit richly persistent errors, relative to blocks requiring stabilization against ***CT*** loads that elicit significantly smaller errors (Figure 4, Error Correction contrast). Because the ***RT*** and ***CT*** perturbations had identical average extensor torque magnitudes (1.2 Nm in both cases), observed differences in this contrast were not due to differences in average torque applied in the two conditions. Consistent with our previous report (Suminski et al., 2007a), active compensation for kinematic performance errors elicits enhanced BOLD signal activation in the cerebello-thalamo-cortical pathways known to be engaged in the feedback control of upper limb movements. Shown in blue are ROIs that exhibited enhanced BOLD signal activation in block conditions having a moving visual cursor, relative to blocks where the cursor was stationary (Figure 4, Visual Motion contrast). As expected, the presence of a moving visual cursor elicited bilateral activations in areas known to process visual motion information and to coordinate movements of the eyes and hands. Broadly speaking, these ROIs include portions of the occipital, posterior parietal, and premotor cortices (Table 1).

**TABLE 1:** Regions exhibiting significant activation in the Stage 1 (block-by-block) contrasts examining error correction and visual motion.

		Talairach coordinates				
	Hem	*X* (mm)	*Y* (mm)	*Z* (mm)	Volume (μl)	Mean T
***Error Correction***						

Precentral gyrus (BA 4,6)	L	–30.2	27.9	51.8	6,798	5.11
Postcentral gyrus (BA 2,3,5)						
Inferior parietal lobule (BA 40)						
Cerebellar cortex (Lobule V, VI)	R	13.7	45.6	–13.1	5,171	5.33
Cerebellar vermis						
Thalamus (VPL, VL, MD, Pulvinar)	L	–16.7	19.3	9.9	3,541	5.07
Cerebellar cortex (Lobule VI)	L	–28.5	46.6	–19.2	1,986	5.73
Inferior parietal lobule (BA 40)	R	57.5	31.9	23.7	1,765	5.12
Thalamus (VPL, VL)	R	17.8	15.5	7.3	1,698	5.03
Medial frontal gyrus (BA 6)	L	–4.6	12.4	48.5	1,216	4.88
Postcentral gyrus (BA 3,40)	R	25.8	34.9	54.1	1,098	5.43
Inferior parietal lobule (BA 40)						
Red nucleus/thalamus	L	–2.2	20.1	–5.6	404	4.98

***Visual motion***						

Sup./Mid./Inf. Occipital Gyrus (BA 18,19)	R	35.4	66.4	14.1	13,268	5.26
Cuneus/precuneus						
Middle temporal gyrus (BA 39)						
Sup./Inf. parietal lobule (BA 7,40)						
Fusiform gyrus (BA 37)						
Superior parietal lobule (BA 7)	L	–28	55.5	45.3	938	5.02
Mid./Inf. occipital gyrus (BA 19,37)	L	–40.5	68.8	3.4	936	4.87
Inferior frontal gyrus (BA 9)	R	46.9	–2.5	25.9	834	4.99
Precentral gyrus (BA 4,6)	L	–40.4	12.7	46.8	797	5.21
Middle occipital gyrus (BA 19)	L	–29.3	80.5	21.6	619	5.08
Cuneus						
Middle frontal gyrus (BA 6)	R	23.9	5.5	45.9	518	4.81
Inferior parietal lobule (BA 40)	R	43.5	33.4	41.7	505	5.40
Pulvinar	R	18	27.6	8.7	382	4.96

*BA, Broadman’s Area; Sup., Superior; Mid., Middle; Inf., Inferior.*

**FIGURE 5:**
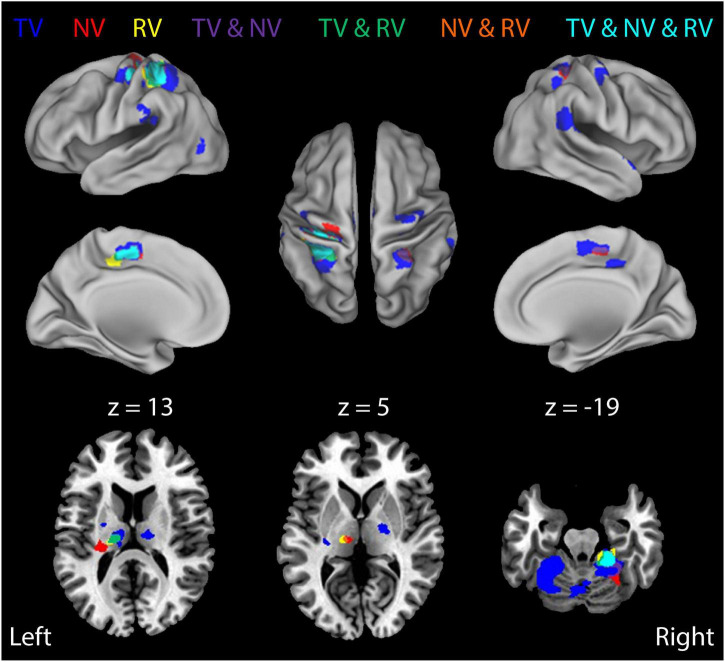
Block-by-block analysis: BOLD signal correlates of ongoing error correction under each of the three visual feedback conditions. Colored regions depict functional activation maps for the study population showing ROIs that exhibited enhanced activation during stabilization against random environmental torques (***RT***) relative to constant torques (i.e., the ***RT*** > ***CT*** contrast) for each of the three different feedback conditions (***TV***: blue; ***NV***: red; ***RV***: yellow). Additional colors indicate regions of overlapping activations (conjunctions) for the three feedback conditions. Neural activities related to error correction are modulated by the presence and fidelity of real-time visual feedback. The results indicate some overlap, but also some differentiation, in regions of activation under the three different feedback conditions.

Next, we investigated how the neural activities related to error correction were modulated by the presence and fidelity of real-time visual feedback (i.e., the interaction between load type and visual feedback condition). We did so by performing a more fine-grained block-wise analysis that involved three additional *t*-test contrasts to visualize how BOLD signal activations in the cerebello-thalamo-cortical pathway vary across the three visual feedback conditions (i.e., ***RTNV-CTNV, RTTV-CTTV*,** and ***RTRV-CTRV***; Table 2). As shown in Figure 5, active stabilization in the absence of visual feedback (i.e., the NV contrast ***RTNV-CTNV***; red ROIs) elicits activations that are largely restricted to regions in cerebello-thalamo-cortical pathways. While activations in this error correction network persist when visual feedback of cursor position is available regardless of fidelity (Figure 5, TV and NV and RV, cyan regions), we found that activations also expand into neighboring areas and appear in new brain regions when veridical visual feedback was available (Figure 5, TV: ***RTTV-CTTV***; blue ROIs). During ***TV*** conditions (relative to ***NV*** conditions), cortical activations in the left primary sensorimotor cortex, cerebellum and bilateral parietal cortex increase in volume encompassing areas traditionally associated with the processing of visuomotor information. Further, additional cortical activations appear in the right premotor cortex, bilateral inferior parietal lobule and left occipital/temporal cortex. Of particular interest are the subcortical activations related to error correction with veridical visual and somatosensory feedback. These areas include the left cerebellar cortex and bilateral ventral lateral nucleus of the thalamus. By contrast, providing incongruent visual feedback (Figure 5, RV: ***RTRV-CTRV;*** yellow ROIs) induced an anterior migration of the stabilization activation volume further into areas known to process somatosensory information, suggesting that subjects exerted additional attentional focus on proprioceptive rather than visual feedback when the two feedback sources were in conflict. This migration (from blue ROIs to the cyan, green, and yellow ROIs) is most prominently observed in the left parietal cortex and left thalamus (*z* = 13).

**TABLE 2:** Regions exhibiting significant activation in the Stage 1 (block-by-block) analysis of proprioceptive errors during wrist stabilization under three different sensory contexts.

		Talairach coordinates				
	Hem	*X* (mm)	*Y* (mm)	*Z* (mm)	Volume (μl)	Mean T
***RTTV v CTTV***						

Postcentral gryus (BA 3)	L	–30.1	31.5	51.8	7,545	5.23
Precentral gyrus (BA 3, 4)						
Inf. parietal lobule						
Sup. parietal lobule (BA 7)						
Precuneus (BA 7)						
Culmen	R	12.2	47.6	–15.1	7,396	5.20
Declive						
Cerebellar lingual						
Nodule						
Culmen	L	–25.8	46.9	–19.8	4,762	5.62
Declive						
Inf. parietal lobule (BA 40)	R	57	31.8	22.9	1,862	5.25
Medial frontal gyrus (BA 6)	L	–2.4	12.2	49	1,846	4.91
Postcentral gryus (BA 40)	R	24.9	36.5	54.2	1,217	5.24
Paracentral lobule (BA 40)						
Thalamus	R	15.7	13.6	9.4	1,049	4.73
Ventral lateral nucleus						
Thalamus	L	–17	17.1	10.7	874	4.62
Ventral lateral nucleus						
Lentiform nucleus						
Inf. parietal lobule (BA 40)	L	–46.3	29.6	24.2	521	4.92
Precentral gyrus (BA 6)	R	30.9	8.4	55.5	429	4.64
Mid. frontal gyrus						
Cerebellar tonsil	L	–23.4	35.9	–44.6	427	5.41
Cerebellar tonsil	R	13.7	48.7	–42.5	422	4.88
Mid. occipital gyrus	L	–37.1	67.5	5.9	396	4.79
Mid. temporal gyrus						
Sup. temporal gyrus (BA 22)	R	53.8	–12.9	–3.8	346	5.49

***RTNV v CTNV***						

Precentral gyrus (BA 4)	L	–29.8	25.4	52.5	4,096	5.02
Postcentral gyrus (BA 3)						
Culmen	R	16.3	44.8	–13.7	2,192	5.29
Mid. occipital gyrus	R	36.7	72.5	12.6	721	5.21
Medial frontal gyrus (BA 6)	L	–5.5	13.1	47.9	556	4.81
Postcentral gyrus	R	26.5	33.9	54.5	362	5.27
Claustrum	L	–24.2	24	14.2	341	4.92
Insula						
Thalamus						
Thalamus	L	–14.5	18.3	6.8	308	4.78
Ventral posterior med. nucleus						

***RTRV v CTRV***						

Precentral gyrus (BA 4)	L	–30.3	29	51.7	3,932	5.20
Inf. parietal lobule						
Postcentral gyrus (BA 40)						
Culmen	R	13.7	42.5	-12.2	3,488	5.38
Thalamus	L	–15.1	18	10.1	911	5.28
Lateral posterior nucleus						
Ventral posterior lateral nucleus						
Paracentral lobule (BA 31)	L	–5.8	12	47.2	373	5.02
Cingulate gyrus (BA 31)						
Paracentral lobule	L	–6.8	23.7	42.9	366	4.96
Cingulate gyrus						

*BA, Broadman’s Area; Sup., Superior; Mid., Middle; Inf., Inferior; Med., Medial.*

#### Stage 2 Analyses: Neural Correlates of Error Correction on a TR-by-TR Time Frame

As mentioned earlier, a previous neuroimaging study has identified distinct neural networks responsible for processing somatosensory-motor errors on long and short time scales during wrist stabilization (i.e., over the full duration of 30 s trials and from one moment to the next; Suminski et al., 2007a). We used a similar approach to probe how the presence and integrity of visual feedback impacts the processing of performance errors on a moment-by-moment basis. Here, residuals from the Stage 1 regression were used as inputs to the Stage 2 TR-by-TR regressions, which sought to identify BOLD signal changes that correlate significantly with performance errors felt proprioceptively [*RMS*_*TR*_(ε_*q*_)] or observed visually [*RMS*_*TR*_(ε_*v*_)]. In contrast to the Stage 1 analyses, the Stage 2 analyses examine neural correlates of information processing specifically related to visual and proprioceptive sensations of stabilization performance errors that fluctuate on a relatively short timescale (i.e., from one 2.5 s TR to the next).

**FIGURE 6:**
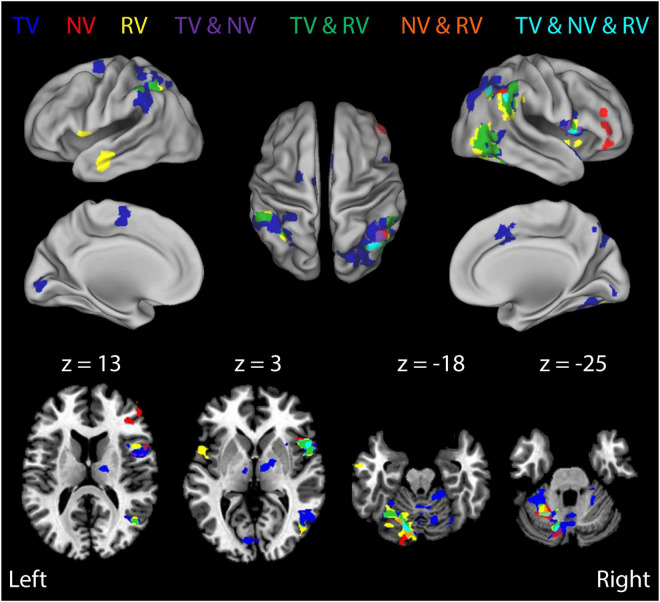
Results of a Stage 2 event-related analysis performed on the BOLD signal residuals from the Stage 1 regressions: TR-by-TR analysis of BOLD signal correlations with wrist angle error *RMS*_*TR*_(ε_*q*_) for each of the three visual feedback conditions. Color coding of functional activation maps is the same as for Figure 5. BOLD signal fluctuations related to TR-by-TR variations in wrist angle error were broadly distributed in sensorimotor areas previously implicated in feedback control of the wrist, including lateral cerebellum, thalamus, posterior parietal cortex, and the supplementary motor area (SMA). Note also the robust activity related to wrist angle error within secondary visual processing areas, even in ***RV*** trials (green- and yellow-shaded regions). By contrast, we observed no significant Stage-2 BOLD signal correlations with visual cursor motion in ***RV*** trials, suggesting that proprioceptive feedback dominated performance in this task, at least during trials with visuo-proprioceptive conflict.

Many regions exhibited BOLD signal changes that were correlated with the time series of somatosensory performance errors *RMS*_*TR*_(ε_*q*_). Figure 6 (and Table 3) presents ROIs exhibiting significant TR-by-TR correlations with *RMS*_*TR*_(ε_*q*_) in each of the three ***RT*** trial conditions with rich, persistent, physical perturbations (***TV***: blue; ***NV***: red; ***RV***: yellow). Additional colors indicate regions of overlapping activations for the three feedback conditions. A comparison of BOLD signal correlations during the ***NV*** condition with the ***TV*** and ***RV*** conditions found that the addition of visual feedback generally caused marked changes in the overall network activity (a drop-out of prefrontal activation as well as dramatically increased activity in bilateral superior/inferior parietal lobule, right superior temporal/middle occipital cortex and left cerebellar cortex). This was particularly true when visual and proprioceptive feedback were congruent; the presence of veridical visual feedback and the neural activities it evoked enabled subjects to enhance wrist stability as shown in Figure 2B. In ***RV*** trials with visuo-proprioceptive conflict, representation of hand stabilization error information was greatly expanded in right hemispheric and left cerebellar regions known to respond preferentially to visual stimuli, but this did not enhance wrist stabilization performance as shown in Figure 2B.

**TABLE 3:** Regions exhibiting significant activation in the Stage 2 (TR-by-TR) analysis of proprioceptive errors during wrist stabilization under three different sensory contexts.

		Talairach coordinates				
	Hem	*X* (mm)	*Y* (mm)	*Z* (mm)	Volume (μl)	Mean T
***TV***						

Fastigium	L	–5.8	52.8	–21.6	8,999	3.78
Denate						
Culmen						
Declive						
Pyramis						
Inf. parietal lobule (BA 40)	R	34.9	52.4	39.1	8,690	3.81
Sup. parietal lobule						
Angular gyrus						
Precuneus						
Inf. parietal lobule (BA 40)	L	–39.7	40.6	40.5	4,498	3.78
Precuneus						
Postcentral gyrus						
Mid. temporal gyrus (BA 37)	R	43.7	57.5	–1.4	4,314	3.76
Inf. temporal gyrus (BA 37)						
Mid. occipital gyrus						
Precentral gyrus	R	47.1	–7.4	8.6	3,824	3.77
Insula						
Inf. frontal gyrus						
Sup. temporal gyrus						
Thalamus	R	6.9	11.1	2.7	1,787	3.77
Cingulate gyrus (BA 32)	R	4.8	–12.7	39.2	774	3.70
Med. frontal gyrus	L	–15.1	4.4	56.1	767	3.77
Cerebellar tonsil	R	23.6	35.1	–41.1	667	3.76
Lingual gyrus	L	–1	77.9	–0.8	651	3.79

***NV***						

Inf. frontal gyrus (BA 46)	R	42.9	–40.4	8.3	1,249	3.72
Declive	L	–14.1	71.6	–21.3	1,129	3.75
Precentral gyrus	R	47.8	–6.5	9.4	1,087	3.75
Inf. parietal lobule (BA 40)	R	42.8	51.2	41.1	911	3.66
Culmen	L	–22.4	53.4	–25.2	542	3.76
Dentate						

***RV***						

Declive	L	–17.3	61.5	–18.9	4,087	3.77
Culmen						
Dentate						
Mid. temporal gyrus (BA 37)	R	45.8	60	0.2	2,707	3.76
Mid. occipital gyrus (BA 37)						
Supramarginal gyrus	R	52.9	39.8	31.1	1,497	3.71
Sup. temporal gyrus						
Inf. parietal lobule (BA 40)						
Insula (BA 13)	R	46.6	–8.5	4.4	1,293	3.76
Sup. temporal gyrus						
Inf. parietal lobule	L	–29.1	46.7	40.5	698	3.73
Angular gyrus	R	35.7	54.3	36.6	681	3.71
Inf. parietal lobule						
Cerebellar tonsil	R	25	31.1	-37.8	617	3.74
Sup. temporal gyrus	L	–47.4	–5.2	1.4	596	3.79
Insula (BA 13)						
Inf. parietal lobule	L	–47.9	34.7	38	571	3.66
Mid. temporal gyrus (BA 21)	L	–54.5	9.9	–14.2	538	3.82

*BA, Broadman’s Area; Sup., Superior; Mid., Middle; Inf., Inferior; Med., Medial.*

By contrast, analysis of the Stage 2 multilinear regression identified no significant correlations with the time series of visuomotor errors [i.e., *RMS*_*TR*_(ε_*v*_)] in either ***RV*** condition. Subjects effectively discounted (or ignored) real-time visual feedback of wrist stabilization errors when visual and somatosensory feedback were in conflict, despite the fact that the mechanical and visual error signals varied in time in similar ways, both in range and spectral content. Taken together, these results suggest that our stabilization task elicited a pattern of interaction between visual and proprioceptive feedback sources that did not conform to the predictions of a MLE model of sensory integration, which given the similar variability of the two feedback signals, would instead predict a more balanced contribution of visual and proprioceptive sources.

**FIGURE 7:**
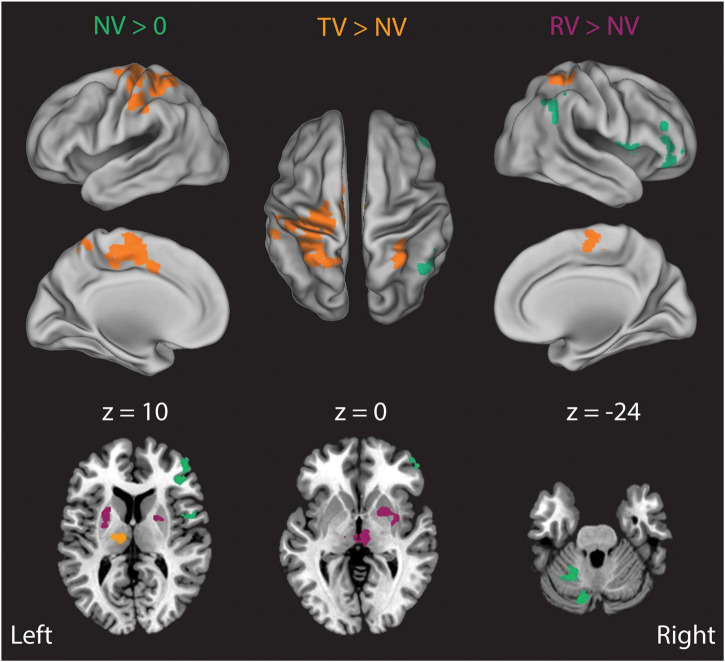
Results of a Stage 2 event-related analysis of how the presence and fidelity of real-time visual feedback influences the moment-by-moment neural processing of proprioceptively-sensed wrist angle errors. In ***NV*** trials (***NV*** > 0, green), BOLD signal correlates of right-hand wrist angle errors were strongest in the left intermediate cerebellum, right posterior parietal cortex, insula and right frontal cortex. Addition of veridical visual feedback (***TV*** > ***NV***, orange) gave rise to a large increase in BOLD signal correlates of proprioceptive errors in the left thalamus, lateral cerebellum, as well as primary sensorimotor, premotor, cingulate motor, and posterior parietal cortices. By contrast, addition of incongruent visual feedback (***RV*** > ***NV***, purple) that was matched in amplitude and bandwidth to actual wrist displacements—but otherwise uncorrelated with them—induced an increase in BOLD signal correlates of actual wrist angle error in the putamen, thalamus and in the red nucleus/ventral tegmental area.

Finally, we examined how the presence and fidelity of visual feedback impacted the processing of somatosensory performance errors by directly comparing Stage 2 analysis BOLD signal correlations in the ***TV*** and ***RV*** conditions to those in the ***NV*** condition (Figure 7). In ***NV*** trials, Stage 2 BOLD signal correlates of right-hand wrist angle errors were strongest in left intermediate cerebellum, and in the right posterior parietal, insula, and frontal cortices (Figure 7, NV > 0, green areas). Adding veridical visual resulted in a dramatic increase in the representation of wrist angle error information in the left primary sensorimotor, premotor, superior/inferior parietal cortices, in the right inferior parietal lobule, and in the left thalamus (ventral lateral, ventral posterior lateral and medial dorsal nucleus) (Figure 7, TV > NV, orange areas). By contrast, somatosensory-error processing in the presence of incongruent random visual feedback yielded an expansion of the representation of wrist angle error information into the bilateral putamen, exterior segment of the globus pallidus, and the right medial dorsal nucleus of the thalamus (Figure 7, RV > NV, purple areas). Activations in these regions suggest their involvement in the context-dependent evaluation of the disparate somatosensory-motor and visuomotor signals and/or the selection of the sensory information feedback channel more likely to facilitate success in the context of the current task. In any case, our analyses reveal an absence of Stage 2 correlates with *RMS*_*TR*_(ε_*v*_) and an abundance of Stage 2 correlates with *RMS*_*TR*_(ε_*q*_) in each of the three ***RT*** trial conditions. This pattern of results argues against the static MLE model of Eq. 1 as a sufficient description of sensorimotor integration for feedback stabilization of the wrist. Instead, the lack of Stage 2 BOLD correlates with visuomotor errors in the ***RV*** conditions is consistent with a model of sensory integration that also performs causal inference (cf. Debats et al., 2017), i.e., where a lack of kinematic correlation between hand and cursor motion operationally segregates the two sources of feedback prior to integration, with only one of them used subsequently for online limb position control.

## Discussion

The primary goal of this study was to examine how the presence and fidelity of visual and proprioceptive feedback impact the neural mechanisms mediating limb stabilization—an important form of mechanical interaction between the body and the environment. To do so, we used a pneumatic robot (Suminski et al., 2007b), functional MR imaging, a long duration wrist stabilization task, and event-related BOLD signal analysis techniques (cf., Suminski et al., 2007a) to elucidate the neural circuits that integrate sensory information from visual and proprioceptive sources to stabilize the wrist against environmental perturbations. In our study, the presence and relative reliability of visual feedback was manipulated, allowing to test whether rules governing integration of visual and proprioceptive information for limb stabilization might conform to models of how the brain uses multisensory feedback for perception (Tillery et al., 1991; Ernst and Banks, 2002; van Beers et al., 2002; Ernst, 2006; Körding et al., 2007; Reuschel et al., 2010; Seilheimer et al., 2014; Debats et al., 2017). In corroboration with a prior neuroimaging study of wrist stabilization in the absence of concurrent visual feedback (Suminski et al., 2007a), we found that wrist stabilization elicited activation in a cerebello-thalamo-cortical circuit known to be engaged in the active feedback control of the upper limb. Relative to a no-vision stabilization condition, the addition of veridical, task-related visual feedback caused activations in the cerebello-thalamo-cortical network to expand (Figures 5–7), ultimately yielding a marked enhancement in behavioral performance (Figure 2B). The intensity and specific loci of expanded activity depended on the fidelity of visual feedback. When incongruous visual feedback was added, additional subcortical activations were observed in areas including the putamen, and thalamus (Figure 7), regions thought to be involved in context dependent action selection and multisensory integration amidst situational uncertainty (Reig and Silberberg, 2014; Wilson, 2014; Robbe, 2018; see also Houk and Wise, 1995). Upon further examination of the correlations between BOLD signal fluctuations and the time series of visual and proprioceptive errors, we found that subjects appeared to rely exclusively on proprioceptive feedback to stabilize the wrist when the fidelity of visual feedback was degraded, even though the mechanical and visual error signals varied similarly in range and spectral content, and despite the fact that objective measures of limb position drifted substantially in many trials without veridical visual feedback. Taken together, these results do not support a model of multisensory integration for action that is governed solely by the MLE rules commonly found to apply to perception. Instead, they likely reflect the action of an early process of causal inference (cf. Körding et al., 2007; Debats et al., 2017), wherein lack of kinematic correlation between hand and cursor motion in the ***RV*** conditions precludes binding of the hand and cursor into a unified object to be manipulated, thereby causing subjects to use just one of the feedback sources (proprioceptive feedback from the hand) for online limb position control.

### Neural Integration of Visual and Proprioceptive Information for Feedback Stabilization of the Wrist

How does the brain integrate the different senses to estimate limb state for the control of stabilization behaviors? The neuromuscular response to perturbation is complex and known to involve at least three primary components: the segmental stretch reflex, long-loop reflex and voluntary responses (Phillips, 1969; Marsden et al., 1972). Our focus here is on the long-loop reflex mechanisms, which consist of neural circuits linking the motor cortex and anterior cerebellum, and which are known to be intimately involved in the closed loop control of limb position (Evarts and Tanji, 1976; Thach, 1978; Evarts and Fromm, 1981; Strick, 1983; Horne and Butler, 1995). In closed loop feedback control, the brain must compare the wrist’s desired position with an estimate of its current state on an ongoing basis, and generate appropriate neuromotor responses to restore the wrist back to its desired position when errors are sensed. How does the brain compose an estimate of the current limb state for use in ongoing feedback control? Previously, we showed that the long-loop pathways involved in feedback control are heavily recruited in the no-vision version of our task when subjects stabilize against random torque perturbations to the wrist, and that it is possible to identify BOLD signal correlates of moment-by-moment changes in performance error using the hierarchical regression technique also employed in the present study (cf. Suminski et al., 2007a). The current results confirm those prior results, and they extend them into two additional sensory contexts involving veridical and incongruent visual feedback conditions (c.f., Figures 5–7). The results demonstrate that the neural processing of somatosensory performance errors depends strongly on the sensory context of the task. These results align well with recent behavioral and electromyographic evidence demonstrating that the long-loop reflex is a flexible, context-dependent mechanism that enables precise feedback control of the limb (Pruszynski et al., 2008, 2011; Nashed et al., 2012; Cluff et al., 2015; Crevecoeur et al., 2016; Ito and Gomi, 2020).

More specifically, results of the Stage 1 analyses show that providing veridical visual information about the position of the hand with respect to the target in our study increased activation in neural circuits typically implicated in visuomotor control (c.f., Vaillancourt, 2003; Vaillancourt et al., 2006) and enabled subjects to reduce the magnitude of stabilization errors relative to the no-vision condition. Improvement in the ability to correct positioning errors using visual feedback, over the course of the 30 s stabilization trial, was mediated by an increase in the volume of activated brain regions in the cerebello-thalamo-cortical pathway (Figure 5; TV) and expansion into other regions supporting visuomotor information processing including the left superior parietal lobule, inferior parietal lobule, cerebellar cortex (lobule IV–VI), right premotor cortex and inferior parietal lobule. By contrast, correcting persistent position errors when visual feedback was unreliable (***RV***) resulted in stabilization performance that was not very different than the no-visual feedback conditions (Figure 2B). This was so even though the condition with unreliable visual feedback still engaged neural activity in the cerebello-thalamo-cortical pathway, albeit to a reduced extent compared to both the veridical and no visual feedback conditions (Figure 5, RV; Table 2). Interestingly, we found that cortical activations in the right premotor/parietal cortex and left cerebellum, which were observed during stabilization with either veridical or no visual feedback, were absent when visual feedback was unreliable. Thus, degrading the reliability of visual information markedly alters the engagement of neural networks normally recruited in visuomotor tasks. These results both support and extend a prior report by Vaillancourt et al. (2006), which examined the effect of intermittent visual feedback on the neural mechanisms of visuomotor control (Vaillancourt et al., 2006). In a grip force control task, they showed that modulating the reliability of visual feedback by reducing its refresh rate reduced the magnitude of BOLD activity in the right premotor and parietal cortex and fully eliminated activity in the cerebellar cortex. This alteration of the neural circuits responsible for correcting persistent errors during periods of reduced sensory fidelity indicates a context-dependent change in control strategy used to integrate sensory information during action (e.g., a switch from one sensory modality to another).

We found further evidence of the context sensitivity of multisensory integration for action in the results of the Stage 2 analyses of BOLD signal correlations with the time series of visual and somatosensory performance errors under the three different sensory feedback conditions. In all cases, we observed BOLD responses related to the TR-by-TR variations in somatosensory performance errors *RMS*_*TR*_(ε_*q*_) to be strongly represented throughout the brain, especially in areas known to process and integrate information from multiple sensory modalities: inferior parietal, superior temporal and lateral occipital cortices (c.f., Beauchamp, 2005; Macaluso, 2006). We found multiple overlapping activations in the right inferior parietal/superior temporal and lateral occipital cortices, where conditions with conflicting sensory feedback (***RV***) were represented more posterior to conditions with veridical information (***TV***). This patchy pattern of activity is similar to previous reports of activity in the superior temporal sulcus during a visual/auditory integration task (Beauchamp et al., 2004). By contrast, we found no activations related to visuomotor errors *RMS*_*TR*_(ε_*v*_) when vision and somatosensation were in conflict, implying that subjects severely discounted (or ignored) visual information in the ***RV*** condition. On the one hand, this outcome was surprising given the similarity in the range and spectral content of the surrogate visual feedback and the applied torque perturbation sequence. Under the assumptions of MLE for multisensory integration (i.e., Eq. 1), we should have expected approximately equal contributions of vision and somatosensation to the feedback stabilization of the wrist given approximately equal amounts of variability in the different feedback signals. On the other hand, the absence of objective kinematic correlation between hand and cursor motions in our task likely weakened any belief that the visual and proprioceptive feedback signals originated from a common source (c.f., Körding et al., 2007; Debats et al., 2017). It is possible that the exclusive selection of proprioception as the preferred source of sensory information may be mediated by neural populations in the striatum, as shown by their increased activity during periods of sensory conflict (Figure 7, purple). These results are consistent with experimental evidence demonstrating multisensory integration in the striatum (Nagy et al., 2006; Reig and Silberberg, 2014; see also Wilson, 2014; Robbe, 2018) and with theoretical work describing the putative role of the basal ganglia as a context detector (Houk and Wise, 1995). Although elucidating the specific mechanism of multisensory integration for limb stabilization would require further refinement of the experimental approach described here (see section “*Limitations and Future Directions”* below), our results nevertheless provide strong support for the idea that the rules governing multisensory integration for action need to account for contextual factors such as the availability of—and kinematic correlation between—the different sensory feedback signals available before and during the task.

### Factors Influencing Sensory Integration for Action

In the INTRODUCTION, we also raised the possibility that the standard MLE model might fail to describe limb state estimation for sensorimotor control in part because the real-time control of action places severe time constraints on the processing of sensory feedback signals that are quite unlike the timing constraints typically imposed in tasks of perceptual decision making. One constraint in tasks requiring fast and accurate movement derives from the fact that sensory feedback of ongoing performance is fleeting; unless feedback is acted on promptly, responses to evolving environmental perturbations can soon become outdated (i.e., unreliable), leading to potential instability in the coupled hand/handle system. Compounding this problem, sensory feedback signals are subject to neural transduction, transmission and processing delays that vary across the different senses: delays in proprioceptive pathways (∼60 ms) are approximately half those in visual pathways (∼120 ms; cf., Cameron et al., 2014).

To address this problem, Crevecoeur et al. (2016) recently proposed a dynamic Bayesian limb state estimation approach that augments the static approach of Eq. 1 with the ability to account for neural information processing delays and noises in the visual and proprioceptive feedback responses. Their model extends a Kalman filter design and yields an optimal state estimate by integrating (delayed) sensory feedback with corresponding prior state estimates for each sensory input. Remarkably, the model predicts that for system with visual delays approaching 100 ms and proprioceptive delays ∼50 ms, the reduction in posterior variance obtained when both vision and proprioception were available was < 10% of the variance obtained with proprioception only. This is much smaller than the reduction expected if the signals were combined based on their variance alone as implied by the model of Eq. 1. As a consequence, simulated kinematic responses to a step-wise mechanical perturbation to the elbow were similar with or without concurrent veridical visual feedback, suggesting that *“when dealing with unpredictable events such as external disturbances, vision plays a secondary role to proprioceptive feedback”* (Crevecoeur et al., 2016). The authors tested the predictions of their model in a series of experiments wherein subjects stabilized their arm against brief elbow torque perturbations presented with and without veridical visual feedback of hand position (as shown by a cursor on a horizontal display screen). Subjects were instructed to follow their hand (or the cursor representing it) with their eyes as they corrected for the perturbation. The authors monitored eye movements as an objective estimate of the subject’s internal estimate of hand location. In agreement with their model, visual feedback-related reductions in arm movement variability were evident toward the later portion of error corrections (relative to their no-vision condition), and the variability of saccadic eye movement endpoints was reduced when visual feedback was also provided. However, the model did not account for the observation that saccade endpoint variability was lowest in a vision-only task that required subjects to track the playback of their prior recorded hand motions. In theory, the variability of a multisensory estimate should be better than that of any unimodal estimate for both the dynamic and static models, suggesting that the visual process *“was not fully contributing following the mechanical perturbations.”* The model also predicted greater actual arm motions in response to the vision-only trials than were observed, prompting the authors to suggest that *“comparing motor responses to visual or mechanical perturbations during reaching may provide additional insight into dynamic multisensory integration.”*

Building on that prior work, we used a long-duration wrist stabilization task and functional MR imaging techniques to infer neural correlates of internal state estimates and the visual and proprioceptive signals that contribute to them on a moment-by-moment (TR-by-TR) basis. If the difference in feedback delays were a primary factor influencing the preferential utilization of somatosensory feedback over visual feedback in our study, as would be suggested by the dynamic Bayesian estimation model, somatosensory feedback should have dominated kinematic performance regardless of whether or not visual feedback were available and veridical. This proposition can be rejected because subjects did in fact leverage veridical visual feedback to improve stabilization performance relative to the NV condition (Figure 2B), and they did so while increasing task-related activity in the cerebello-thalamo-cortical pathway and other visuomotor support areas (Figures 5–7). Instead, it is probable that additional important factors influencing multisensory integration pertain to the coherence between the available sensory signals (cf., Debats et al., 2017) and prior expectations as to whether the hand and cursor move together as a common controlled object (c.f., Körding et al., 2007). Because the robot’s physical perturbations stimulated a rich set of proprioceptors sensitive to mechanical stimuli (including stretch receptors embedded in muscle bodies, force-sensitive Golgi tendon organs, and pressure-sensitive mechanoreceptors in the glabrous skin of the hand), *RMS*_*TR*_(ε_*q*_) was bound to be highly correlated with each of these afferent signals regardless of sensory context in this study. By contrast, the visual feedback signal *RMS*_*TR*_(ε_*v*_) had high coherence with the somatosensory signals in the ***TV*** condition and low coherence with those signals in the ***RV*** condition. A lack of cross modal sensory coherence in the ***RV*** conditions could have been a trigger that caused subjects to discount the cursor feedback as irrelevant to the task at hand: i.e., physically stabilizing the wrist. A neural mechanism for such context-dependent gating is suggested by the BOLD signal activations observed in the basal ganglia and thalamus in the ***RV*** > ***NV*** contrast shown in Figure 7.

### Limitations and Future Directions

This study has several limitations. One limitation derives from our experimental design, which only included the two extreme visual feedback conditions (***TV*** and ***RV***) in addition to the ***NV*** control condition. Our design did not include intermediate blends of congruent and incongruent visual feedback, which would have allowed to test whether the lack of BOLD signal correlation with *RMS*_*TR*_(ε_*v*_) in the current study was due to the fact that this signal was uncorrelated with the physical consequences of perturbation, as shown in Figure 3. A future study could address this limitation by requiring wrist stabilization in the presence of visual feedback θ_*vision*_(*t*) that could be variably masked by bandlimited Gaussian noise θ_*surrogate*_(*t*) as in:


(5) $$\theta_{vision}\left(t\right)=\lambda \theta_{hand}\left(t\right)+\left(1-\lambda \right)\theta_{surrogate}\left(t\right).$$

Here, λ is a weighting factor determining the extent to which visual cursor motion corresponds to actual motion of the wrist θ_*hand*_(*t*) vs. bandlimited noise. Under the hypothesis that visual stimuli are discounted below some threshold of cross-modal coherence, one might expect to observe BOLD signal correlates with θ_*surrogate*_(*t*) in brain regions involved in the low-level processing of moving visual stimuli when λ takes on moderate values, but to not observe such correlates when λ approaches extreme values of 0 or 1. However, other hypotheses are possible; if slow visual feedback is not really involved in the moment-by-moment formation of feedback responses to performance errors but is instead used to calibrate (or center) the faster proprioceptive feedback corrections about the desired goal posture, then one might expect to observe no BOLD signal correlates with θ_*surrogate*_(*t*) for any value of λ.

Another limitation derives from the fact that the data we present were collected on a 1.5T MR scanner, which limited our image resolution and whole brain image capture rate. A significant benefit of using functional MR imaging in this study (rather than some other imaging technique such as electroencephalography, EEG) includes the ability to image the whole brain for neural correlates of signals of interest such as somatosensory and visual performance errors. If it were possible to repeat the study on a higher resolution scanner, the resulting data would undoubtedly have improved spatial and temporal resolution relative to those presented here. However, it is unlikely that any of our main conclusions would change, in part because the neural events of interest in functional MR imaging studies are commonly low-pass filtered both in space (with a blurring filter to accommodate inter-subject anatomical differences in across-subjects analyses) and in time (with a γ-variate or related hemodynamic response function to account for the sluggish physiological hemodynamic response). As shown above, our data have sufficient resolution to detect changes in the Stage 1 block-wise analyses of BOLD signals related to the different environmental load and sensory feedback conditions (Table 1 and Figures 4, 5). The data show that relative to the ***NV*** control condition, wrist-angle error-related BOLD signals expand in the cerebello-thalamo-cortical pathways known to contribute to feedback stabilization when veridical visual feedback is added, and they shift to include brain regions involved in context detection and action selection when incongruent visual feedback is added. Our data also suffice to identify Stage 2 BOLD signal correlates of wrist angle errors over a much shorter, TR-by-TR time frame (Table 2 and Figures 6, 7) riding on top of the signals described in the Stage 1 analysis. Improved temporal resolution would undoubtedly improve the statistical power of the Stage 2, TR-by-TR regressions, which could in turn improve sensitivity to BOLD signal correlates of *RMS*_*TR*_(ε_*v*_), the ***RV*** errors sensed visually. However, no degree of improved spatial or temporal resolution would change the observation that BOLD signal correlates of error signals sensed proprioceptively far outpower BOLD correlates of ***RV*** errors sensed visually; this degenerate outcome was rather unexpected because the magnitude and spectral characteristics of the visual feedback was similar in the ***TV*** and ***RV*** conditions. The fact that visual errors elicit no measurable BOLD signal correlates during wrist stabilization in the ***RV*** case argues against the idea that multisensory integration for limb stabilization is adequately described by the form of MLE model often posed for multisensory integration for perceptual decision making tasks, even as updated to account for differing sensory feedback delays. Instead, our findings bolster the idea that an early stage of sensorimotor control—prior to integration—involves discrete decisions: the binding of kinematically correlated feedback signals into a unified object to be controlled and the segregation/suppression of uncorrelated signals that are assumed to be task irrelevant. Further research is needed to clarify which contextual factors impact causal inference and multisensory integration for perception, cognition, and action.

## Data Availability Statement

The raw data supporting the conclusions of this article will be made available by the authors, without undue reservation.

## Ethics Statement

The studies involving human participants were reviewed and approved by the Marquette University IRB Medical College of Wisconsin IRB. The patients/participants provided their written informed consent to participate in this study.

## Author Contributions

AS and RS contributed to conception and design of the study, wrote the first draft of the manuscript, and wrote sections of the manuscript. AS collected the data. AS and RD performed the statistical analysis. All authors contributed to manuscript revision, read, and approved the submitted version.

## Author Disclaimer

The opinions, findings, and conclusions, or recommendations expressed are those of the author(s) and do not necessarily reflect the views of the National Science Foundation.

## Conflict of Interest

The authors declare that the research was conducted in the absence of any commercial or financial relationships that could be construed as a potential conflict of interest.

## Publisher’s Note

All claims expressed in this article are solely those of the authors and do not necessarily represent those of their affiliated organizations, or those of the publisher, the editors and the reviewers. Any product that may be evaluated in this article, or claim that may be made by its manufacturer, is not guaranteed or endorsed by the publisher.
